# Anti-CD3 Fab Fragments Enhance Tumor Killing by Human γδ T Cells Independent of Nck Recruitment to the γδ T Cell Antigen Receptor

**DOI:** 10.3389/fimmu.2018.01579

**Published:** 2018-07-09

**Authors:** Claudia Juraske, Piyamaporn Wipa, Anna Morath, Jose Villacorta Hidalgo, Frederike A. Hartl, Katrin Raute, Hans-Heinrich Oberg, Daniela Wesch, Paul Fisch, Susana Minguet, Sutatip Pongcharoen, Wolfgang W. Schamel

**Affiliations:** ^1^Department of Immunology, Faculty of Biology, University of Freiburg, Freiburg, Germany; ^2^Centre for Biological Signalling Studies (BIOSS), University of Freiburg, Freiburg, Germany; ^3^Center for Chronic Immunodeficiency (CCI), Medical Center – University of Freiburg, Faculty of Medicine, University of Freiburg, Freiburg, Germany; ^4^Department of Microbiology and Parasitology, Faculty of Medical Science, Naresuan University, Phitsanulok, Thailand; ^5^Spemann Graduate School of Biology and Medicine (SGBM), University of Freiburg, Freiburg, Germany; ^6^Department of Pathology, Faculty of Medicine, University of Freiburg, Freiburg, Germany; ^7^University Hospital “José de San Martin”, University of Buenos Aires, Buenos Aires, Argentina; ^8^Institute of Immunology, Christian-Albrechts University of Kiel, Kiel, Germany; ^9^Division of Immunology, Department of Medicine, Faculty of Medicine, Naresuan University, Phitsanulok, Thailand; ^10^Research Center for Academic Excellence in Petroleum, Petrochemical and Advanced Materials, Faculty of Science, Naresuan University, Phitsanulok, Thailand; ^11^Centre of Excellence in Medical Biotechnology, Faculty of Medical Science, Naresuan University, Phitsanulok, Thailand

**Keywords:** γδ T cells, T cell antigen receptor, tumor, Nck, activation, signaling, Fab fragments, AX-024

## Abstract

T lymphocytes expressing the γδ T cell receptor (γδ TCR) can recognize antigens expressed by tumor cells and subsequently kill these cells. γδ T cells are indeed used in cancer immunotherapy clinical trials. The anti-CD3ε antibody UCHT1 enhanced the *in vitro* tumor killing activity of human γδ T cells by an unknown molecular mechanism. Here, we demonstrate that Fab fragments of UCHT1, which only bind monovalently to the γδ TCR, also enhanced tumor killing by expanded human Vγ9Vδ2 γδ T cells or pan-γδ T cells of the peripheral blood. The Fab fragments induced Nck recruitment to the γδ TCR, suggesting that they stabilized the γδ TCR in an active CD3ε conformation. However, blocking the Nck-CD3ε interaction in γδ T cells using the small molecule inhibitor AX-024 neither reduced the γδ T cells’ natural nor the Fab-enhanced tumor killing activity. Likewise, Nck recruitment to CD3ε was not required for intracellular signaling, CD69 and CD25 up-regulation, or cytokine secretion by γδ T cells. Thus, the Nck-CD3ε interaction seems to be dispensable in γδ T cells.

## Introduction

T cells are part of the adaptive immune system and can be divided into αβ and γδ T cells, depending on the T cell antigen receptor (TCR) they express. Whereas most αβ TCRs recognize peptides presented by MHC molecules, γδ TCRs recognize stress-induced self-antigens (1, 2), lipids or pyrophosphates that are secreted by microbes or overproduced in tumor cells (3–7).

The main subset of γδ T cells in human blood is Vγ9Vδ2, which accounts for 2–10% of all T cells. The Vγ9Vδ2 TCR recognizes self and foreign non-peptidic phosphorylated small organic compounds of the isoprenoid pathway, collectively termed phosphoantigens (8–12). It is known that Vγ9Vδ2 T cells are also stimulated by certain tumor cells, such as the Daudi B cell lymphoma (13), which most likely expresses high levels of phosphoantigens (14). Alternatively, γδ T cells recognize cell surface molecules that are differentially expressed on transformed solid tumors or lymphomas and leukemias (7, 15). An enhanced production of phosphoantigens in transformed cells can be further increased by therapeutically administered nitrogen-containing bisphosphonates, such as zoledronate, which inhibit the farnesyl pyrophosphate synthase of the isoprenoid pathway (16, 17). Several studies demonstrated that a repetitive stimulation of γδ T cells *in vivo* is necessary to reduce tumor growth (18–20). While sustained stimulation of Vγ9Vδ2 γδ T cells by phosphoantigens or nitrogen-containing bisphosphonates often leads to their exhaustion, bispecific antibodies provide a newly tool to target γδ T cells to antigens expressed by tumor cells and enhanced their cytotoxic activity (19, 21–23). Although the exact molecular mechanism leading to phosphoantigen recognition is a matter of debate (24, 25), this recognition is clearly mediated by cognate interaction with the Vγ9Vδ2 TCR.

T cell antigen receptors consist of a clonotypic TCRαβ or TCRγδ heterodimer, and the CD3δε, CD3γε, and CD3ζζ dimers. TCRαβ and TCRγδ bind to the antigen and the CD3 chains transduce the signal of antigen binding into the cell by phosphorylation of the tyrosines in their cytoplasmic tails by Src-family kinases. Consequently, the tyrosine kinase ZAP70 can bind to phosphorylated CD3 and the signal of ligand binding is transmitted further to intracellular signaling cascades, such as Ca^2+^ influx and the Ras/Erk pathway, ultimately resulting in the activation of the T cell. This includes the execution of the cytotoxic activity to kill infected or tumor cells, up-regulation of CD69 and CD25, as well as secretion of cytokines.

How antigen binding to the TCR is communicated to the cytosolic tails of CD3 is not well understood. The αβ TCR is in equilibrium between two reversible conformations: the antigen-stabilized active CD3 conformation and the resting conformation adopted by non-engaged TCRs (26–28). The active CD3 conformation is stabilized by peptide-MHC or anti-CD3 antibody binding to the αβ TCR (29, 30), and it is absolutely required (but not sufficient) for TCR activation (27, 30–32). This active CD3 conformation is defined by the exposure of a proline-rich sequence (PRS) in CD3ε that then binds to the SH3.1 domain of the adaptor protein Nck [SH3.1(Nck)] (26, 33). Blocking the CD3ε–Nck interaction by the small molecule inhibitor AX-024 or by other means diminished ligand-induced CD3 phosphorylation and downstream signaling events (34–36). Shifting to the active CD3 conformation is necessary for αβ TCR triggering, however, it is not sufficient (30, 37). Fab fragments of anti-CD3 antibodies stabilize the active conformation, but are unable to elicit biochemical signals leading to T cell activation (30, 38, 39). In addition, antigen-induced αβ TCR clustering and/or phosphatase exclusion are required, most likely to elicit stable phosphorylation of the ITAMs and thus, T cell activation (30, 37, 40).

How antigen binding to the γδ TCR is transmitted to the cytosolic tails of CD3 is even more obscure. Antigen binding to TCRγδ failed to expose the CD3ε’s PRS, in sharp contrast to the αβ TCR, but efficiently activated the γδ T cell (41). Artificial induction of the active conformation by binding the anti-CD3ε antibody UCHT1 to the γδ TCR enhanced the cytotoxic activity of human γδ T cells against a pancreatic tumor cell line (41). Whether Nck is recruited to γδ TCRs in the natural or the UCHT1 enhanced activity and whether this plays a role in the increased tumor killing is to date unknown.

Here, we used expanded γδ T cells from human peripheral blood of healthy donors and show that UCHT1 and Fab fragments of UCHT1 lead to the recruitment of Nck to the γδ TCR. Further, we stimulated the γδ T cells with B cell lymphomas and demonstrate that UCHT1 Fab fragments increase the tumor killing by the γδ T cells and that Nck binding to the γδ TCR is not involved in this tumor killing.

## Materials and Methods

### Expansion of Human γδ T Cells

Informed consent for the performed studies was obtained from the donors in accordance with the Declaration of Helsinki and Institutional Review Board approval from the University of Freiburg Ethics Committee (412/9). Human peripheral blood mononuclear cells were isolated from healthy donors by using a Ficoll–Hypaque gradient. Cells were adjusted to 10^6^ cells/ml and cultured in RPMI 1640 supplemented with 10% fetal calf serum (FCS) and antibiotics.

To expand Vγ9Vδ2 γδ T cells, cells were stimulated with 2.5 µM zoledronate and 50 U/ml rIL-2 (Novartis). Additionally, rIL-2 was added every 2 days over a culture period of 21 days. After 14 days the purity of expanded γδ T cells was analyzed by flow cytometry and was >95% Vγ9Vδ2 T cells.

To expand different γδ T cell subsets, cells were stimulated with 1 µg/ml concanavalin A and rIL-2 and rIL-4 (both 100 U/ml) were added to the cell suspensions. Additionally, rIL-2 and IL-4 were added every 3–4 days over a culture period of 21 days. After 14 days γδ T cells were enriched by negative isolation using the human TCRγ/δ^+^ T Cell Isolation Kit (Miltenyi Biotech). Cultures were composed of 20–40% Vδ1^+^ and 60–80% Vδ2^+^ T cells and the purity of the enriched γδ T cells was evaluated by flow cytometry. Cultures with a purity >95% were used for the experiments.

### ^51^Cr Release Assay

Daudi or Raji tumor cells (5,000 cells/well in a 96-well plate) were incubated with 50 µl ^51^Cr for 1 h at 37°C. After labeling, cells were washed two times and the cell concentration was adjusted to 5 × 10^4^ cells/ml in RPMI 1640 supplemented with 10% FCS, antibiotics, and 50 U/ml rIL-2 (Novartis). The assay was performed in U bottom 96-well plate, with an effector (γδ T cell) to target (Daudi or Raji cells) ratio of 12.5:1, in triplicates and with six replicates for spontaneous (^51^Cr release from target cells in medium alone) and maximum release (^51^Cr release from target cells lysed with the detergent Trition X-100). The required amount of effector cells (6.25 × 10^4^ cells/well) was harvested and the concentration was adjusted to 1.25 × 10^6^ cells/ml in RPMI 1640 supplemented with 10% FCS and 50 U/ml rIL-2 (Novartis). To the labeled tumor cells the expanded γδ T cells were added, and left untreated (−) or treated with 5 µg/ml UCHT1, 3.33 µg/ml Fab, or 3.33 µg/ml Fab_red_ (to obtain equimolar amounts). Additionally, DMSO or different concentrations of the inhibitor AX-024 (36) solubilized in DMSO were added. Cells were incubated at 37°C and 5% CO_2_ for 5 h. After incubation, 50 µl of the supernatant was transferred into ^51^Cr filter plates (Lumaplate). The plates were measured in a microplate scintillation γ-ray counter and data were acquired in counts per minute (cpm). Cytotoxicity was calculated according to the formula: specific lysis (%) = (experimental cpm − spontaneous cpm): (maximum cpm − spontaneous cpm) × 100.

### SH3.1(Nck) Pull Down Assay

Zoledronate-expanded γδ T cells were starved for 1 h at 37°C in RPMI 1640 without FCS and left unstimulated or stimulated with 5 µg/ml anti-CD3 antibody UCHT1. Cells were lysed in lysis buffer containing 20 mM Tris–HCl pH 8, 137 mM NaCl, 2 mM EDTA, 10% glycerol, protease inhibitor cocktail, and 0.3% Brij96V. Subsequently, insoluble material was removed by centrifugation. Postnuclear fractions were incubated with glutathione beads coupled to GST-SH3.1(Nck) at 4°C as described (26). Beads, including the bound proteins, were washed and proteins separated by non-reducing SDS-PAGE. Western blotting was performed with anti-CD3ζ and anti-GST antibodies.

### Preparation of Fab and Fab_red_ Fragments

UCHT1 Fab fragments were prepared using the Pierce^®^ Fab Micro Preparation Kit from Thermo Fisher Scientific, which uses the enzyme papain to cleave the complete UCHT1 antibody and protein A coupled beads to purify the Fab fragments. The purified Fab fragments were analyzed by SDS-PAGE and Coomassie staining. To generate reduced Fab fragments (Fab_red_) the purified Fab fragment was incubated with 10 mM dithiothreitol for 30 min at room temperature. Afterward, 1 mM iodoacetamide was added and incubated for further 30 min at room temperature. The Fab_red_ were then immediately used. Purity of Fab and Fab_red_ was further tested by their inability to induce Ca^2+^ influx in T cells.

### Flow Cytometry

Concanavalin A expanded γδ T cells were incubated with 5 µg/ml UCHT1, 3.33 µg/ml Fab, or 3.33 µg/ml Fab_red_ or left untreated at 37°C and 5% CO_2_ for 5 h. Cells were washed two times and stained with APC-labeled anti-mouse IgG antibody on ice (Southern Biotech). The labeled cell suspension was analyzed by the Gallios™ flow cytometer and the data were analyzed with FlowJo software.

Jurkat and G8 γδ TCR-expressing Jurkat cells were stained with APC-labeled anti-mouse γδ TCR (GL3; eBioscience). Cells were analyzed as described above.

### Measurement of Ca^2^^+^ Influx

Five million cells were resuspended in 1 ml RPMI 1640 medium supplemented with 1% FCS in the presence or absence of 10 nM AX-024, antibiotics, and labeled in the dark with 0.1% pluronic acid, 2.6 µM Fluo-3 AM, and 5.5 µM Fura Red AM (Life Technologies) for 45 min at 37°C. The stained cells were washed and kept on ice in the dark until the measurement. For calcium influx, cells were diluted 1:20 with pre-warmed medium and maintained at 37°C during the event collection on a CyAn ADP flow cytometer (Beckman Coulter). Baseline fluorescence was monitored for 1.5 min, then the stimuli were added as indicated (the Fab and Fab_red_ fragments were added 30 s earlier to ensure binding to the γδ TCR when the other stimuli were added). The stimulation was recorded for further 5 min. Data were analyzed with the FlowJo software.

### Intracellular Staining for Phospho-ZAP70 and Phospho-Erk

Peripheral blood mononuclear cells were isolated from healthy donors using a Ficoll–Hypaque gradient. T cells were obtained by negative isolation using the Pan T cell Isolation Kit (Miltenyi Biotech). Cells were taken in RPMI 1640 supplemented with 10% FCS and were rested for 1 h at 37°C in the presence or absence of different concentrations of the inhibitor AX-024 (36). Cells were left unstimulated or stimulated with 10 µg/ml anti-CD3 antibody UCHT1 for 2 min or 5 min and were fixed with 2% paraformaldehyde for 30 min on ice. Subsequently, cells were permeabilized with 87.7% methanol for 30 min on ice and stained with rabbit anti-phospho-ZAP70 (Cell Signaling) or rabbit phospho-Erk (Cell Signaling) overnight. Next, cells were stained with biotin-labeled anti-CD3 (UCHT1; BioLegend), PE-labeled anti-γ/δTCR antibodies (Life Technologies), and DyLight-labeled anti-rabbit IgG (Thermo Scientific) and subsequently with eFluor 450-labeled Streptavidin (eBioscience). Cells were measured by flow cytometry and gated on CD3- and TCRγδ-positive cells for analysis.

### *In Situ* Proximity Ligation Assay (PLA)

Cells were grown on diagnostic microscopic slides (Thermo Scientific). They were left unstimulated, stimulated with 5 µg/ml UCHT1, 3.33 µg/ml Fab, or 3.33 µg/ml Fab_red_ and simultaneously treated with 10 nM AX-024 at 37°C for 5 min. Cells were then fixed with 4% paraformaldehyde, permeabilized with 0.5% saponin, and blocked with blocking solution. Subsequently, cells were co-incubated with the goat anti-CD3ε (M20ε, Santacruz) and a rabbit anti-Nck1 antibody (Cell Signaling). PLA between the CD3ε and Nck1 molecules was performed with the Duolink kit according to the manufacturer’s instructions (Olink Bioscience), resulting in red fluorescence signals. Cell nuclei were stained with DAPI. A confocal microscope (C2, Nikon) was used for imaging and analysis. The number of the PLA signal dots was scored with the BlobFinder program (Uppsala University).

### CD25 and CD69 Up-Regulation

To each well of a U bottom 96-well plate 2 × 10^4^ Daudi or Raji tumor cells, 2.5 × 10^5^ expanded γδ T cells, and 5 µg/ml UCHT1, 3.33 µg/ml Fab, 3.33 µg/ml Fab_red_, or medium (unstimulated) were given. In addition, the cells were left untreated or treated with different concentrations of AX-024. The plate was incubated at 37°C and 5% CO_2_ for 18 h. After incubation, the supernatants were kept at −80°C to quantify the amounts of TNFα and IFNγ (see below). Cells were stained with APC-labeled anti-CD25 (eBioscience) or APC-labeled anti-CD69 (Life Technologies) together with PE-labeled anti-γ/δTCR antibodies (Life Technologies). Cells were analyzed by flow cytometry gating on γδTCR-positive cells.

### TNFα and IFNγ Secretion

The concentrations of TNFα and IFNγ in the culture supernatants were measured by standard enzyme linked immunosorbent assay (ELISA).

### Statistical Analysis

Data are represented as mean ± SEM or ± SD. All differences between experimental groups were analyzed with the Student’s *t*-test. Significant differences were considered when the *p* values were less than 0.05. (n.s. = non significant, *p < 0.05, **p < 0.01, ***p < 0.001, and ****p < 0.0001). *n* refers to the number of independently performed experiments.

## Results

### The Anti-CD3ε Antibody UCHT1 Enhances Tumor Killing by Human γδ T Cells

**Figure 1:**
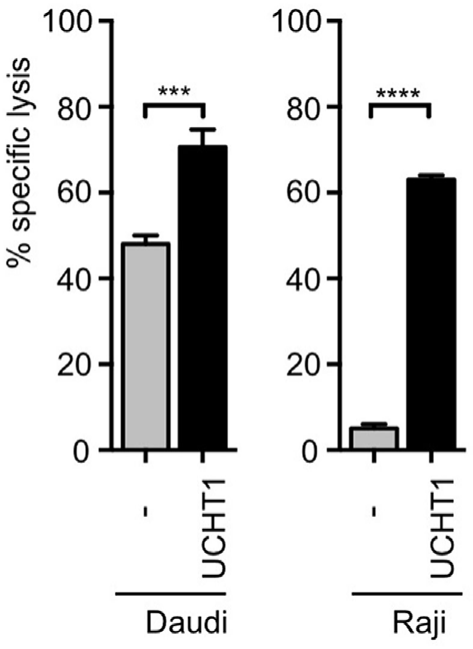
UCHT1 increased tumor killing by γδ T cells. ^51^Cr-labeled Daudi and Raji cells were incubated with zoledronate-expanded human γδ T cells without (−) or with the addition of 5 µg/ml UCHT1 using an effector to target (T cell to tumor cell) ratio of 12.5:1. After 5 h of co-culture, the amount of released ^51^Cr was measured in the supernatant by γ-ray spectroscopy. Data represent mean ± SD of triplicate wells (*n* = 3). Significance was determined by unpaired *t*-test, two-tailed between untreated γδ T cells and treated samples.

Human short-term expanded Vγ9Vδ2 γδ T cells can kill pancreatic ductal adenocarcinoma Panc89 cells *in vitro* (21, 23), which was enhanced by co-incubation with the anti-CD3ε antibody UCHT1 (41). In order to test target cells from different origins, we used now the ^51^Cr-labeled human B cell lymphoma lines Daudi or Raji (13) and incubated them with zoledronate-expanded peripheral blood human γδ T cells in the presence or absence of UCHT1. Release of ^51^Cr to the cell culture supernatant is a measure for the lysis of the tumor cells and was quantified with a scintillation γ-ray counter. Approximately 40% of the Daudi and 5% of the Raji cells were specifically killed by the γδ T cells in the absence of exogenously added antibody (Figure 1). The addition of the UCHT1 antibody strongly enhanced tumor killing by the γδ T cells reaching 75% of specific killing for both cell lines (Figure 1).

### Using Its SH3.1(Nck) Domain, Nck Is Recruited to the UCHT1-Stimulated γδ TCR

**Figure 2:**
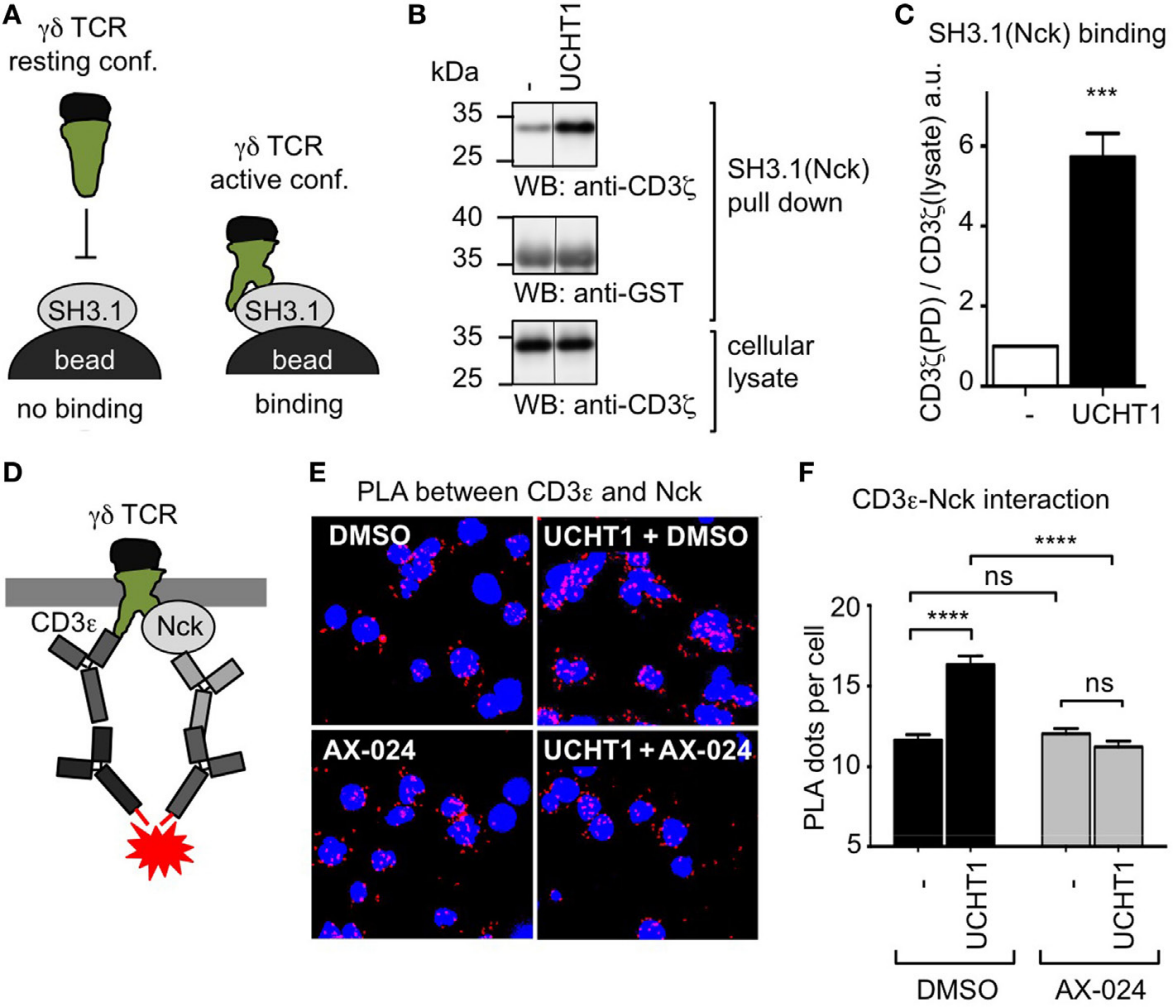
Using its SH3.1(Nck) domain Nck binds to the UCHT1-stimulated γδ T cell antigen receptor (TCR). **(A)** Schematic of the SH3.1(Nck) pull down assay. **(B)** Zoledronate-expanded γδ T cells were left untreated (−) or stimulated with 5 µg/ml UCHT1 at 37°C for 5 min. The cellular lysates were incubated with SH3.1(Nck)-coupled beads and bound proteins separated by SDS-PAGE along with aliquots of the cellular lysate. Western blotting was done using anti-CD3ζ and anti-GST antibodies. **(C)** The normalized ratio of the band intensities of CD3ζ to GST in the SH3.1(Nck) pull down is plotted. Data of three independent experiments were used to calculate the mean ± SD; statistics was done by two-tailed *t*-test. **(D)** Schematic of the *in situ* proximity ligation assay (PLA) using anti-CD3ε and anti-Nck antibodies. **(E)** Close proximity between the TCR and Nck was detected by *in situ* PLA. Zoledronate-expanded γδ T cells were either left untreated (−) or treated with 5 µg/ml UCHT1 in the absence or presence of 10 nM AX-024 at 37°C for 5 min. After fixation and permeabilization, PLA was performed using the primary antibodies goat anti-CD3ε (M20) and rabbit anti-Nck1, and the corresponding secondary antibodies. Nuclei were stained with DAPI. **(F)** The corresponding quantification of the red PLA dots and the mean ± SEM is displayed; statistics was done by two-tailed *t*-test. For each condition 500 cells were analyzed. Three independent experiments were performed (*n* = 3).

UCHT1 stabilizes the γδ TCR in the active CD3 conformation, in which the CD3ε PRS is exposed (41). Since only the exposed PRS binds to SH3.1(Nck), PRS exposure can be measured with the SH3.1(Nck) pull down assay (Figure 2A). To test whether the UCHT1-bound γδ TCR can bind to SH3.1(Nck) in our zoledronate-expanded γδ T cells, we incubated the cells with or without UCHT1 and lysed them. Performing a pull down assay using SH3.1(Nck)-coupled beads, we found that UCHT1 increased the amount of γδ TCR that bound to the beads (Figures 2B,C). This indicated that SH3.1(Nck) can bind to the γδ TCR and that UCHT1 stabilizes the γδ TCR in the active CD3 conformation.

To date it is unknown whether endogenous Nck is recruited to the γδ TCR upon stimulation. To test this, we used the *in situ* PLA. PLA is a technique that allows visualization of the close proximity between endogenous proteins in fixed cells by a red fluorescent dot (42). Recently, we established the PLA to quantify the proximity of Nck with the αβ TCR using anti-CD3ε and anti-Nck antibodies (33). Here we applied this assay to γδ T cells. To this end, we incubated expanded human γδ T cells with UCHT1 and performed PLA (Figure 2D). Indeed, CD3ε–Nck proximity was increased in UCHT1-stimulated cells compared to unstimulated cells (Figures 2E,F) suggesting that Nck is indeed recruited to the γδ TCR upon UCHT1 binding.

To test whether endogenous Nck uses its SH3.1 domain to bind to the CD3ε PRS in the γδ TCR, we made use of the small molecule inhibitor AX-024. In αβ T cells AX-024 was shown to specifically bind to SH3.1(Nck) and hence block the SH3.1(Nck)–PRS interaction (36). Using αβ T cells as a control, we made sure that PLA is a suitable assay to test the inhibition of the SH3.1(Nck)–PRS interaction mediated by AX-024 (Figure S1 in Supplementary Material). Next, we incubated the γδ T cells with UCHT1 in the absence or presence of 10 nM AX-024. Indeed, the CD3ε–Nck proximity was reduced to background levels after AX-024 treatment (Figures 2E,F), indicating that the PRS–SH3.1 interaction is necessary for the recruitment of Nck to the γδ TCR upon UCHT1 binding. This finding was corroborated with a PLA experiment in which CD3 phosphorylation was blocked using the Src kinase inhibitor PP2 (Figure S2 in Supplementary Material). Indeed, Nck was recruited to the γδ TCR in the presence of PP2, suggesting that an SH2(Nck)-phospho-CD3 interaction was not required.

### Preparation of Pure UCHT1 Fab Fragments

**Figure 3:**
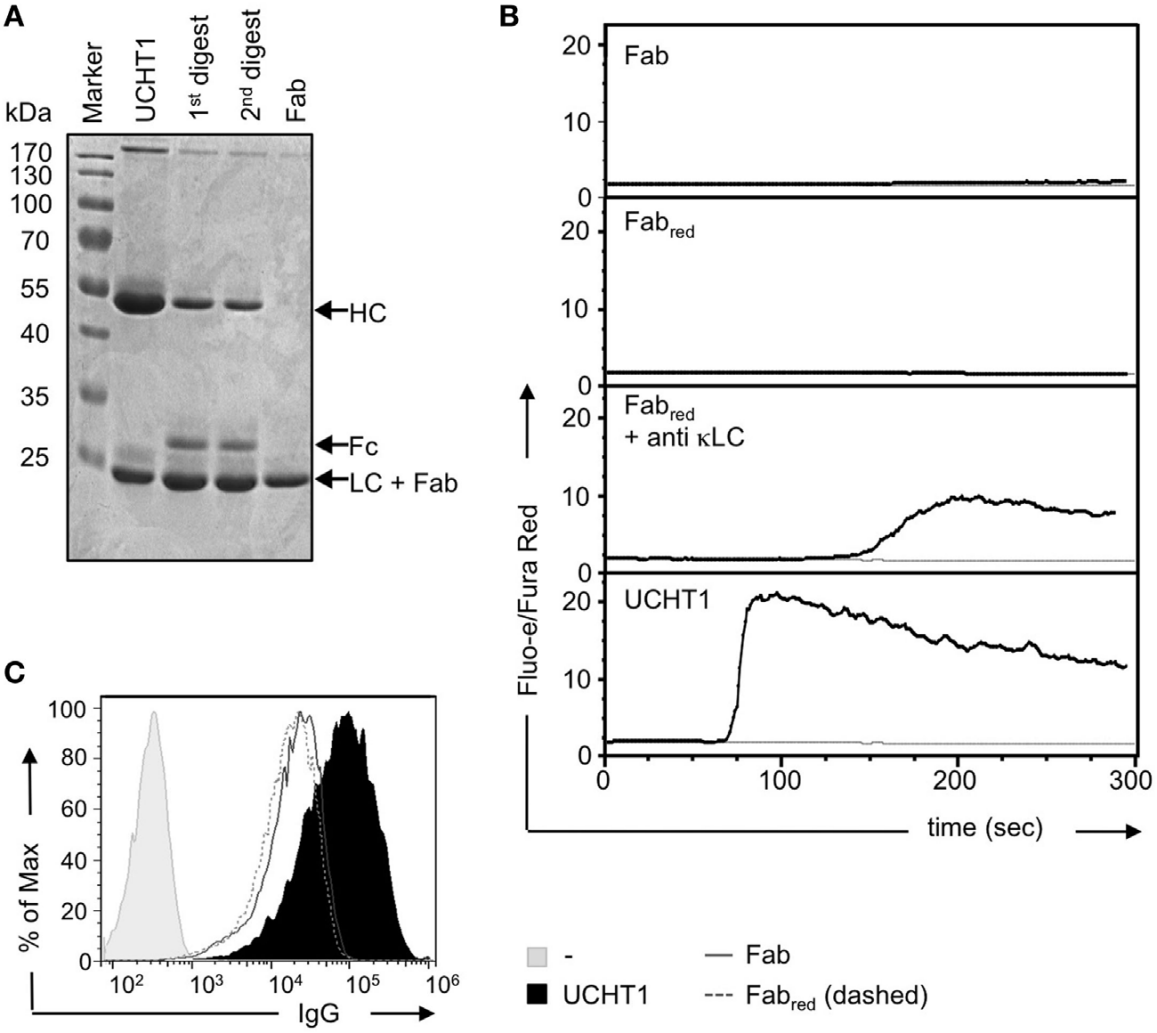
Preparation of UCHT1 Fab fragments. **(A)** The anti-CD3ε antibody UCHT1 was digested with papain using a kit from Thermo Fisher Scientific. The Fab fragments were purified with protein A coupled beads. The complete antibody (lane 1), the first and second digestions (lanes 2 and 3), and the purified Fab fragment (lane 4) were separated by reducing SDS-PAGE followed by Coomassie staining (*n* > 3). **(B)** Jurkat Vγ9Vδ2 cells were labeled with Fluo-3 AA and Fura Red AM and the baseline of cellular Ca^2+^ was measured by flow cytometry for 1 min. The indicated reagents were then added and Ca^2+^ levels were measured for additional 6 min (black lines). The gray lines represent baseline Ca^2+^ level without addition of any stimuli (*n* > 3). **(C)** Concanavalin A expanded γδ T cells were stained with UCHT1, Fab, Fab_red_, or left untreated, followed by an APC-labeled anti-mouse IgG antibody as a secondary reagent. Fluorescence intensities were quantified by flow cytometry (*n* > 3).

Complete anti-CD3ε antibodies bind bivalently to the TCR leading to T cell activation, whereas Fab fragments of the same antibodies bind monovalently and fail to activate T cells (30, 37–39). The use of complete anti-CD3ε antibodies in therapeutical settings might have the drawback of unspecific polyclonal T cell activation. Thus, we aimed here to investigate whether UCHT1 Fab fragments that only bind monovalently to the TCR might enhance tumor killing by human γδ T cells in the absence of undesired unspecific T cell activation. To this end, we generated pure Fab fragments by cleaving UCHT1 antibodies with papain and subsequently purifying the Fab fragments using the Pierce Fab Micro Preparation kit. The complete UCHT1 antibody before and after digestion and the purified Fab fragments were analyzed by reducing SDS-PAGE and Coomassie staining (Figure 3A). We found that the complete immunoglobulin heavy chain (HC) at 50 kDa was not detectable in the Fab fragments, but the light chain (LC) was traceable at 24 kDa (lane 4). As the variable and CH1 parts of the HC have the same size as the LC, they are not visible as an additional band in SDS-PAGE (Figure 3A, Fab).

We found earlier that very small amounts of *F*(ab′)_2_ fragments might contaminate the Fab preparation. They are not detectable by SDS-PAGE and Coomassie staining, but due to their cross-linking ability they can lead to TCR activation in functional assays (30). *F*(ab′)_2_ fragments can be reduced to Fab fragments by the use of dithiothreitol (DTT) followed by quenching of DTT with iodoacetamide (30). To test for the presence of small amounts of contaminating UCHT1 or UCHT1 *F*(ab′)_2_ fragments in the Fab preparation, we incubated our reagents with TCRαβ-negative Jurkat T cells expressing the human Vγ9Vδ2 TCR (43, 44) and measured calcium influx into the cytosol as a read-out for TCR activity. The unreduced Fab fragments either did not induce any or just a very small increase in cytosolic calcium (Figure 3B, upper panel), indicating that some preparations were contaminated with tiny amounts of *F*(ab′)_2_. However, the reduced Fab fragments never induced any calcium response (Figure 3B, second panel). As a control, the reduced Fab fragment cross-linked by anti-κ LC antibodies, resulted in calcium influx, indicating that the reduced Fab fragments were functional. Treatment of the cells with the complete UCHT1 antibody resulted in the strongest calcium response (Figure 3B, lowest panel). From now on, UCHT1 Fab fragments will be called “Fab” and the reduced fragments “Fab_red_.”

By using staining and flow cytometry, we show that Fab and Fab_red_ can equally well bind to human γδ T cells (Figure 3C).

In conclusion, Fab_red_ preparations contained functional Fab fragments that are purely monovalent and failed to crosslink and activate the TCR.

### UCHT1 Fab Fragments Enhance Tumor Killing by Human γδ T Cells

**Figure 4:**
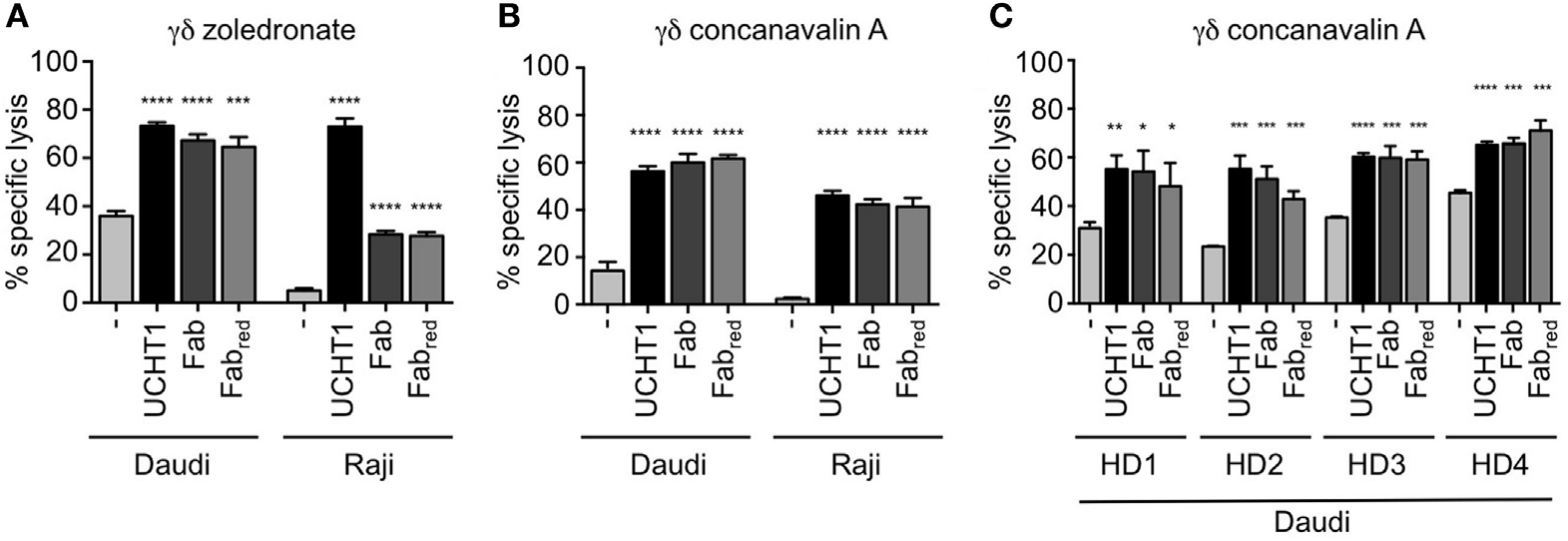
Fab and Fab_red_ fragments enhanced tumor killing by γδ T cells. ^51^Cr-labeled Daudi and Raji cells were incubated with zoledronate **(A)** or concanavalin A **(B)** expanded γδ T cells in triplicates without (−) or with 5 µg/ml UCHT1, 3.33 µg/ml Fab, or 3.33 µg/ml Fab_red_ (these concentrations were chosen to obtain equimolar amounts of the different reagents). The effector to target ratio was 12.5:1. After 5 h of co-culture, the amount of released ^51^Cr was measured in the supernatant by γ-ray spectroscopy (*n* = 3). **(C)** Concanavalin A expanded γδ T cells from four different donors (HD1, 2, 3, and 4) were used in a chromium-release assay using Daudi cells as target as in **(A)** (*n* = 1). Data represent mean ± SD of triplicate wells. Significance was determined by unpaired *t*-test, two-tailed between untreated γδ T cells and UCHT1, Fab or Fab_red_-treated samples.

Next, we tested whether Fab and Fab_red_ enhance tumor killing by γδ T cells. To this end, we co-cultured zoledronate-expanded γδ T cells with Daudi or Raji cells as in Figure 1. Both, Fab and Fab_red_ increased killing of the tumor cells (Figure 4A). In Daudi cells, which were more susceptible to the cytotoxic activity of the γδ T cells, Fab, and Fab_red_ were almost as active as UCHT1. In Raji cells, which were only killed to 5% by γδ T cells, Fab and Fab_red_ enhanced the cytotoxic activity, so that 30% of the cells were killed.

Zoledronate and IL-2 specifically expand human Vγ9Vδ2 γδ T cells (16, 23). γδ T cells can also be expanded from human peripheral blood using the lectin concanavalin A, IL-4, and IL-2, resulting in cultures containing both, Vδ1 and Vδ2 γδ T cells (45). Concanavalin A, IL-4, and IL-2 expanded γδ T cells (here called concanavalin A expanded γδ T cells) also killed Daudi and Raji cells, and this activity was increased by UCHT1, Fab, and Fab_red_ (Figure 4B). This effect was not donor-specific, since the enhanced tumor killing mediated by UCHT1, Fab, and Fab_red_ was also observed when using concanavalin A expanded γδ T cell from blood of four different healthy donors (HD, Figure 4C).

### Nck Is Recruited to the γδ TCR Upon Binding to Fab and Fab_red_

**Figure 5:**
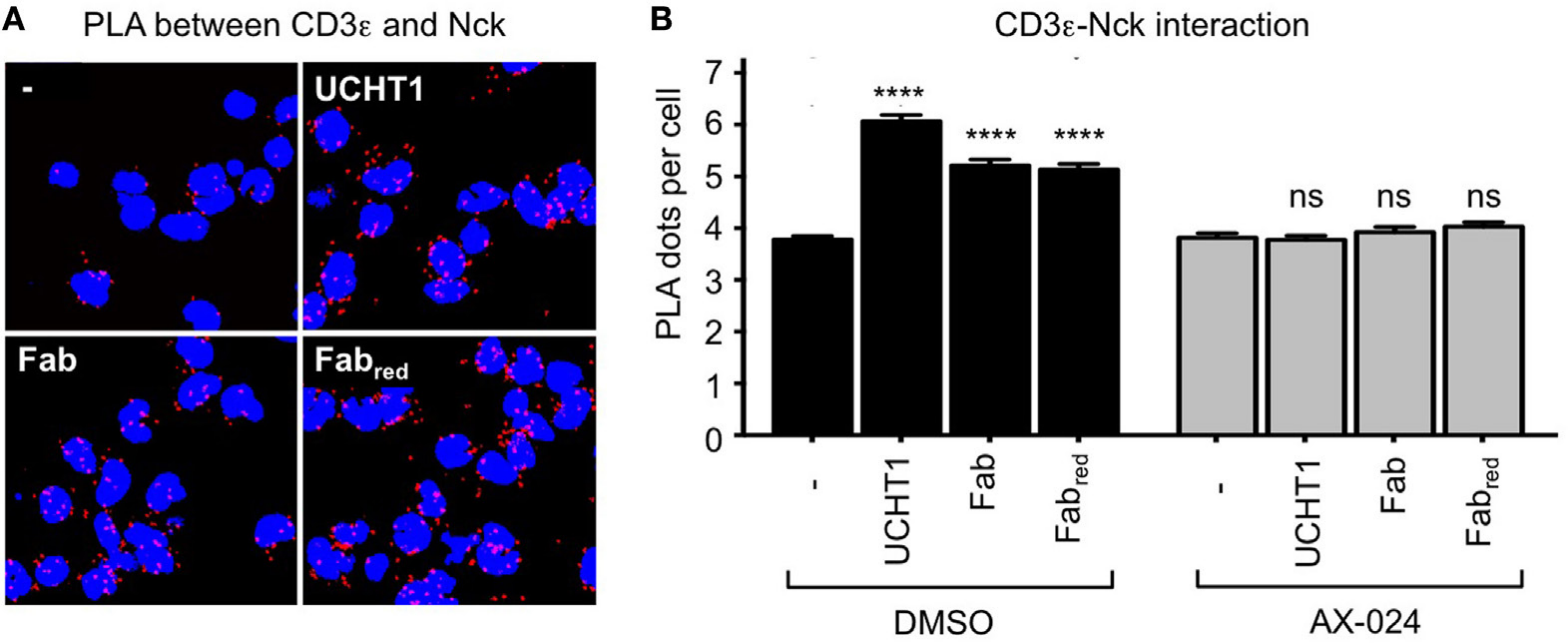
UCHT1 Fab induced the recruitment of Nck to the γδ T cell antigen receptor. **(A)** The proximity ligation assay as in Figure 2 was performed using 5 µg/ml UCHT1, 3.33 µg/ml Fab, and 3.33 µg/ml Fab_red_ in the absence or presence of 10 nM AX-024. **(B)** The data of **(A)** were analyzed as in Figure 2F; 700 cells were analyzed per condition. Two independent experiments were performed (*n* = 2).

Fab and Fab_red_ do not cross-link the γδ TCR, but might enhance tumor killing by stabilizing the active CD3 conformation of the γδ TCR—just as the complete UCHT1 antibody does (Figure 2). Hence, we used PLA to test whether Fab and Fab_red_ induced the recruitment of Nck to the γδ TCR. Indeed, treatment of the γδ T cells with Fab and Fab_red_ resulted in an increase in TCR–Nck proximity (Figures 5A,B). Again, the incubation with the small molecule inhibitor AX-024 reduced Nck binding to the γδ TCR to background levels. This finding suggests that binding of Fab and Fab_red_ to the γδ TCR artificially stabilizes the active CD3 conformation and results in the recruitment of Nck to the γδ TCR using the SH3.1(Nck) domain.

### Tumor Killing by γδ T Cells Is Independent of Nck Recruitment to the γδ TCR

**Figure 6:**
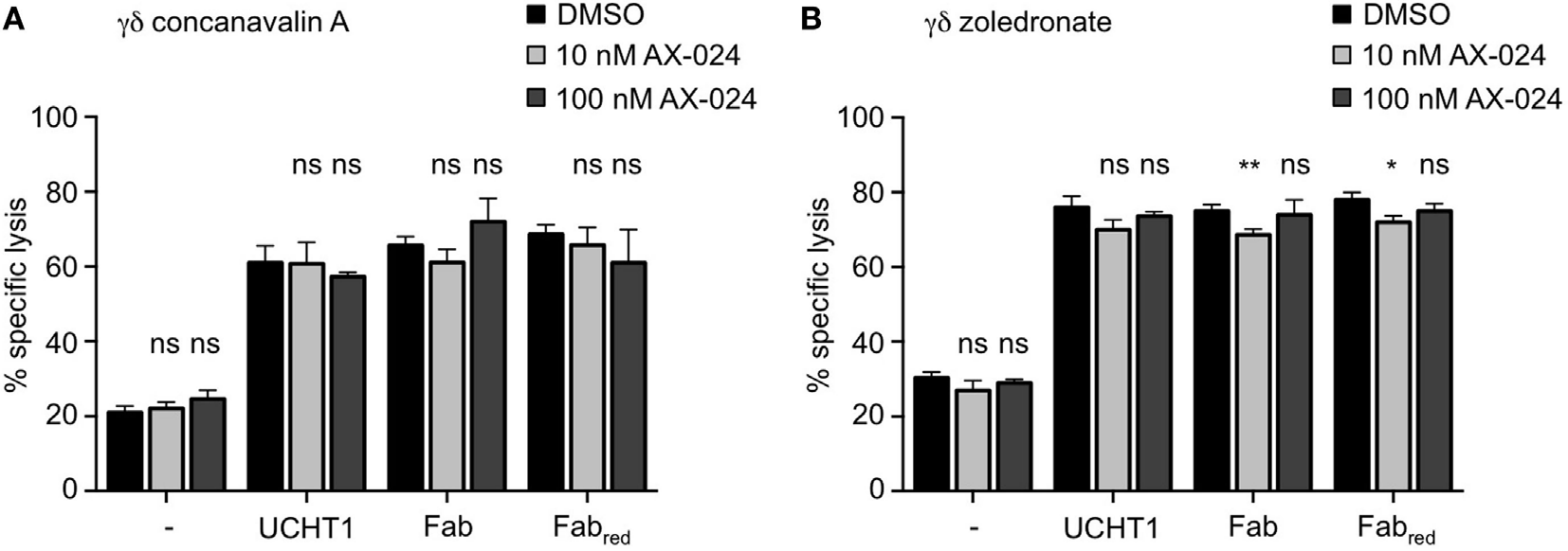
Tumor killing by γδ T cells is independent of Nck recruitment to the γδ T cell antigen receptor. ^51^Cr-labeled Daudi cells were incubated with concanavalin A **(A)** or zoledronate **(B)** expanded γδ T cells without (−) or with 5 µg/ml UCHT1, 3.33 µg/ml Fab, or 3.33 µg/ml Fab_red_. In addition, 10 or 100 nM of AX-024 was included or not. The effector to target ratio was 12.5:1. After 5 h of co-culture, the amount of released ^51^Cr was measured in the supernatant by γ-ray spectroscopy. Data represent mean ± SD of triplicates (*n* = 3). Significance was determined by unpaired *t*-test, two-tailed between untreated samples and samples treated with AX-024.

We next analyzed whether Nck recruitment to the γδ TCR upon UCHT1, Fab, or Fab_red_ treatment was involved in γδ T cell activation mediated by tumor cells, and for the cytotoxic activity of γδ T cells. Concanavalin A expanded γδ T cells (Figure 6A) or zoledronate-expanded γδ T cells (Figure 6B) were incubated with Daudi cells in the presence or absence of UCHT1, Fab, or Fab_red_ with or without AX-024 to block the PRS–SH3.1 interaction. Two concentrations of AX-024 were used, 10 nM AX-024 as in Figure 5 and 100 nM AX-024. All three anti-CD3 reagents significantly enhanced tumor killing by the γδ T cells. AX-024 did not disturb the γδ T cell-mediated “basal” tumor cell killing (without anti-CD3 reagents). Importantly, AX-024 did also not influence the enhanced killing activity in the presence of the anti-CD3 reagents for the concanavalin A expanded γδ T cells (Figure 6A). In case of the zoledronate-expanded γδ T cells (Figure 6B) very small reductions with 10 nM, but not with 100 nM AX-024, can be detected. Hence, we concluded that AX-024 did also not diminish tumor killing for the zoledronate-expanded γδ T cells.

These findings indicate that Nck recruitment to the γδ TCR is dispensable for the cytotoxic activity of γδ T cells stimulated by tumor cells.

### Activation of γδ T Cells Is Independent of Nck Recruitment to the γδ TCR

**Figure 7:**
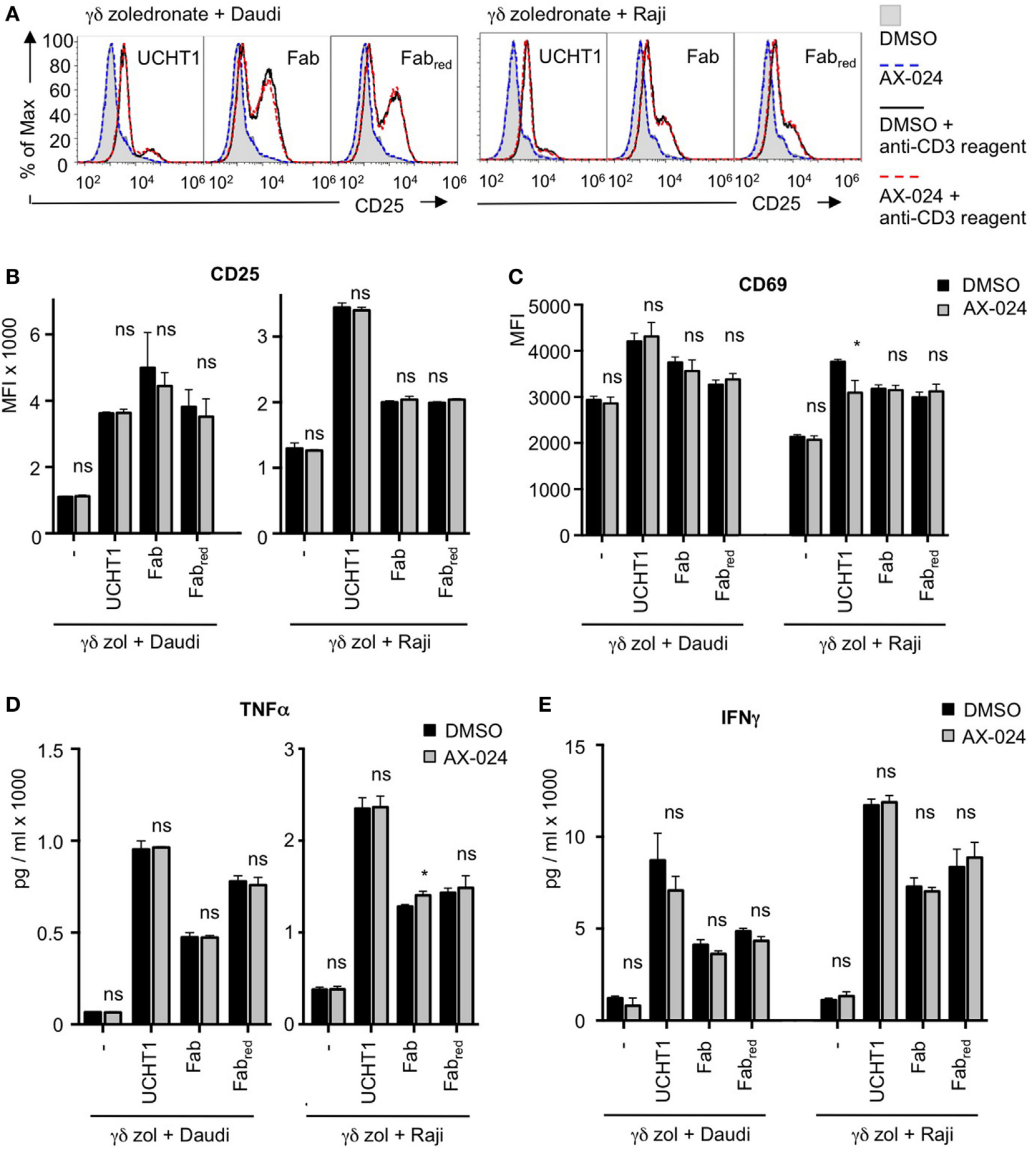
The up-regulation of activation markers and cytokines is independent of Nck recruitment to the γδ T cell antigen receptor (TCR). Daudi and Raji cells were incubated with zoledronate-expanded γδ T cells without (−) or with 5 µg/ml UCHT1, 3.33 µg/ml Fab, or 3.33 µg/ml Fab_red_. In addition, 10 nM AX-024 was included or not. The γδ T cell to target cell ratio was 12.5:1. The cells were co-cultured at 37°C and 5% CO_2_ for 18 h. Subsequently, cells were either stained with anti-CD25 and anti-γ/δTCR **(A,B)** or with anti-CD69 and anti-γ/δTCR **(C)** antibodies. Fluorescence intensities were quantified by flow cytometry **(A)** and the mean fluorescence intensity is displayed **(B,C)**. Data represent mean ± SD of triplicates (*n* = 3). Significance was determined by unpaired *t*-test, two-tailed between untreated samples and samples treated with AX-024. **(D,E)** The experiments were performed as in **(A)**. After co-culturing of the cells for 18 h, the supernatants were harvested. TNFα **(D)** and IFNγ **(E)** concentrations were quantified by enzyme linked immunosorbent assay.

In addition to cytotoxic activity, γδ T cell activation involves up-regulation of the expression of the high affinity IL-2 receptor CD25 and of the activation marker CD69 (46). Next, we sought to analyze whether Fab can also enhance these activation events in γδ T cells and whether Nck binding to the γδ TCR was required for that. Zoledronate-expanded γδ T cells were stimulated with Daudi or Raji cells in the presence or absence of UCHT1, Fab, or Fab_red_ with or without AX-024. As with tumor cell killing, all three anti-CD3 reagents enhanced CD25 (Figures 7A,B) or CD69 (Figure 7C) up-regulation by the γδ T cells. Blocking the γδ TCR–Nck interaction with AX-024 did neither affect the expression of CD25 nor of CD69 independently of the anti-CD3 reagents (Note that the decrease of UCHT1 enhanced CD69 expression by AX-024 was not seen in other experiments).

We have also tested whether AX-024 impacts on CD69 up-regulation in fresh, naive γδ T cells from human blood. As with the expanded cells, AX-024 did not change the extent of CD69 expression when the cells were stimulated with UCHT1 (Figure S3 in Supplementary Material).

Furthermore, γδ T cells secrete pro-inflammatory cytokines, such as TNFα and IFNγ, upon activation by tumor cells (47). Finally, we show that TNFα and IFNγ production induced by Daudi or Raji cells was enhanced by UCHT1, Fab, or Fab_red_ (Figures 7D,E). AX-024 neither diminished TNFα and IFNγ secretion in the absence nor in the presence of the anti-CD3 reagents.

Together, our data indicate that UCHT1, Fab, or Fab_red_ binding to the γδ TCR enhances the tumor cell-induced activation of γδ T cells. Although Nck is recruited to the anti-CD3 bound γδ TCR, this recruitment seems to be dispensable for γδ T cell activation events, such as cytotoxicity, CD25, and CD69 up-regulation, as well as TNFα and IFNγ secretion.

### Fab Fragments Enhance Intracellular Signaling Independent of the Nck-γδ TCR Interaction

If Nck recruitment to the γδ TCR is not involved in tumor killing and the up-regulation of activation markers, it might also not be required for the γδ TCR induced induction of intracellular signaling. To test this, we stimulated fresh human γδ T cells with UCHT1 in the absence or presence of AX-024 and measured the phosphorylation of the kinases ZAP70 and Erk by flow cytometry (Figures S4A,B in Supplementary Material). As expected, γδ TCR stimulation increased the amount of phospho-ZAP70 and phospho-Erk. Importantly, treatment of the cells with AX-024 did not influence the extent of ZAP70 or Erk phosphorylation, indicating that recruitment of Nck to the γδ TCR was not required for the induction of signaling by the UCHT1-stimulated γδ TCR.

**Figure 8:**
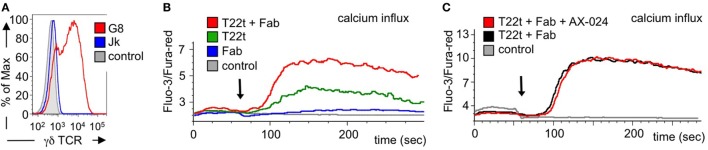
Calcium signaling is enhanced by Fab fragments and does not require the Nck-γδ T cell antigen receptor (TCR) interaction. **(A)** G8 γδ TCR-expressing Jurkat cells were stained with the γδ TCR-specific antibody GL3 and analyzed by flow cytometry. As controls, the GL3-stained parental Jurkat cells and unstained G8 γδ TCR-expressing cells are shown (*n* > 3). **(B)** Jurkat cells expressing the G8 γδ TCR were stimulated with the G8 ligand T22 tetramers (T22t), UCHT1 Fab, or a combination of both T22t and Fab. Intracellular calcium was measured using the dyes Fluo-3 and Fura-red by flow cytometry. Calcium influx is depicted as the median ratio of Fluo-3 to Fura-red fluorescence over time. As a negative control, steptavidin was added to the cells. The arrow indicates addition of the stimuli (*n* = 3). **(C)** The experiment was performed as in **(C)** with the difference that cells without and with 10 nM AX-024 were stimulated with the combination of T22t and Fab. As a control, PBS was added to the cells (*n* = 3).

Next we asked whether Fab fragments would also increase signaling by an antigen-triggered γδ TCR. In order to use a cognate ligand for the γδ TCR, we switched to a different γδ TCR system, namely the G8 γδ TCR where a clearly defined ligand, namely MHC class I-like T22, can be used (48, 49). In fact, stimulation of G8 γδ TCR-expressing cells with soluble T22 tetramers leads to T cell stimulation without stabilizing the CD3 conformational change at the γδ TCR (41). Here, we expressed the G8 γδ TCR in Jurkat T cells, similar to as we did with a chimeric γδ TCR in a Jurkat-derived cell (27). Indeed, the G8 γδ TCR was expressed on the surface of the Jurakt cells (Figure 8A).

Next, we stimulated the G8 γδ TCR-expressing Jurkat cells with soluble T22 tetramers and measured the amount of intracellular calcium as a signaling read-out. As expected, the Fab fragments hardly induced calcium signaling downstream of the γδ TCR, whereas T22 tetramers did (Figure 8B). Importantly, adding Fab to the T22 tetramers augmented the calcium response, most likely by stabilizing the γδ TCR in the active CD3 conformation. The same result was obtained when using Fab_red_ fragments (Figure S5A in Supplementary Material).

To test whether the enhanced calcium response was sensitive to the recruitment of Nck to the γδ TCR, we stimulated the G8 γδ TCR-expressing Jurkat cells with the T22 tetramers and Fab in the absence or presence of AX-024. Clearly, AX-024 treatment did reduce the calcium response (Figure 8C), suggesting that the Nck–γδ TCR interaction was not required for the enhanced calcium signaling. The same result was obtained when using Fab_red_ fragments (Figure S5B in Supplementary Material).

Together our data suggest that Fab fragments enhance signaling *via* the γδ TCR by stabilizing the active CD3 conformation, independent of the Nck–γδ TCR interaction.

## Discussion

The potential use of cytotoxic γδ T cells in immunotherapy against cancer is particularly attractive. Since γδ T cells are independent of antigen presentation by MHC class I and of the presence of mutated epitopes, they are ideal effectors against tumors with low mutation loads. Cytotoxic γδ T cells display afferent responses and have been recognized as the best favorable prognosis marker for solid tumors when infiltrated immune cells were analyzed (50). This suggested that γδ T cells play an important role in the defense against tumors in human patients. Therefore, γδ T cells are currently tested as cellular reagents in cancer immunotherapy clinical trials (51–53).

We have previously shown that the tumor killing activity of γδ T cells can be increased by addition of the anti-CD3ε antibody UCHT1. However, UCHT1 is a potent T cell activating agent due to its intrinsic capability to induce cross-linking of TCRs and to stabilize the active CD3 conformation (27, 41). Thus, UCHT1 activates all T cells (αβ and γδ), regardless of their specificity, possibly resulting in an unspecific polyclonal T cell response and a potential life-threatening cytokine storm. These drawbacks profoundly limit the use of UCHT1 as a therapeutical agent to enhance tumor cell killing by cytotoxic γδ T cells that recognize tumor antigens by their γδ TCR. Here, we aimed to explore the use of UCHT1 Fab fragments as an alternative to enhance tumor killing by γδ T cells. UCHT1 Fab fragments do not activate αβ TCRs by themselves due to their monovalent binding (30, 38, 39) and here we show that this is also the case with the γδ TCR. Importantly, we demonstrate that UCHT1 Fab fragments significantly boosted tumor cell killing by γδ T cells, suggesting the use of UCHT1 Fab fragments as specific co-stimulation agents in γδ T cell immunotherapy approaches.

All three ligands that were previously tested for the γδ TCR (Daudi and phosphoantigens for the human Vγ9Vδ2 TCR and T22 tetramers for the murine G8 TCR) did not stabilize the active CD3 conformation, as defined by the exposure of the CD3ε PRS (41). In contrast, UCHT1 did stabilize the γδ TCR in its active conformation (41). In addition, UCHT1 binds simultaneously to two TCRs and thereby crosslinks γδ TCRs, leading to γδ TCR and T cell activation. Thus, by using UCHT1, it is not possible to distinguish whether cross-linking of TCRs or stabilization of the CD3 active conformation is the event enhancing tumor killing by γδ T cells. To answer this mechanistic question, we used here monovalent UCHT1 Fab fragments, which only have one binding site per molecule and, therefore, do not cross-link TCRs. Our data show that UCHT1 Fab fragments also enhanced the cytotoxic activity of human γδ T cells. Thus, a cross-linking activity is not required to boost γδ T cell cytotoxic activity. We next tested whether UCHT1 Fab fragments also stabilize the active γδ CD3 conformation and thereby, induce the recruitment of Nck to the CD3ε PRS. The treatment of the γδ T cells with the Fab fragments or with UCHT1 led to the recruitment of Nck to the γδ TCR. This induced recruitment was abrogated in the presence of the inhibitor AX-024, which blocks the interaction of the CD3ε PRS with SH3.1(Nck) (36). And indeed SH3.1(Nck) can bind to the CD3ε PRS in γδ TCRs [(41) and this study]. These data provide strong mechanistic evidence demonstrating that Nck binds to the γδ TCR *via* the CD3ε PRS upon stabilization of the active CD3 conformation, like in the αβ TCR (26, 33).

Human Vγ9Vδ2 γδ T cells modestly killed the B cell lymphoma lines Daudi and Raji. This killing was enhanced in the presence of UCHT1 Fab. Daudi and other tumor cells express high levels of phosphoantigens (14), which together with butyrophilin 3A1 most likely constitute (part of) the ligand for the Vγ9Vδ2 TCR (24, 25). Thus, the γδ TCR was likely to be bound to the natural phosphoantigen/butyrophilin 3A1 ligand in our experimental settings. This potential binding stimulated the γδ TCR, but without stabilizing the γδ TCR in its active CD3 conformation (41). Here, we show that, in addition to stimulation by the natural ligands, the γδ TCR was stabilized in its active conformation when we used the UCHT1 Fab fragments. This treatment not only enhance the tumor cell killing, but also the activation of γδ T cells as seen by augmented CD69, CD25, IFNγ, and TNFα expression. Enhanced up-regulation of CD69 and CD25 was also seen with the complete UCHT1 antibody ( (41) and this study). However, increased production of IFNγ and TNFα by UCHT1 was not seen earlier (41) and this could be due to differences in the cells used (expanded γδ T cells versus a γδ T cell clone). Our data thus support the idea that enforcing the γδ TCR to adopt the active CD3 conformation in the presence of its natural ligand might generate a quantitatively or/and qualitatively distinct set of activation signals that ultimately enhance γδ T cell activation.

One mechanism to promote such distinct signals might have been the recruitment of Nck to the γδ TCR. We found, however, that Nck recruitment was dispensable for the enhanced activation of the γδ T cells in the presence of the Fab fragments or the complete antibody. This suggests that a so far unknown effect of the active CD3 conformation in the γδ TCR increases γδ T cell activity. One possibility might be enhanced phosphorylation of the CD3 tails on tyrosines in analogy to the αβ TCR (27, 28), which might lead to a stronger T cell stimulation. Indeed, the kinase ZAP70 that binds to doubly phosphorylated CD3 subunits (54) and is recruited to the γδ TCR (55) was phosphorylated upon stimulation of γδ T cells with UCHT1. Since this phosphorylation was independent of Nck recruitment, it might be a downstream effect of the active CD3 conformation that we were looking for.

ZAP70 is important to trigger signaling cascades in αβ T cells, such as the Erk and calcium pathways (54), and these pathways are also triggered by stimulation of the Vγ9Vδ2 TCR by phosphoantigens or anti-CD3 antibodies (41, 55–59). Here, we show that calcium influx stimulated by the cognate ligand–γδ TCR interaction was increased upon stabilization of the active CD3 conformation. This is in line with our earlier finding that trapping the γδ TCR in the resting CD3 conformation by using CD3ε mutants [CD3εK76T and CD3εC80G (31, 32)] reduced γδ TCR calcium signaling (41). Similarly UCHT1 enhanced phosphorylation of Erk [(41) and this study], and this again was independent of Nck recruitment to the γδ TCR. Since the Erk pathway is involved in the antitumor activity of Vγ9Vδ2 T cells (56), an increase in phospho-Erk might explain the enhanced tumor killing when the active CD3 conformation was stabilized. In conclusion, stabilization of the active CD3 conformation in a ligand-triggered γδ TCR leads to enhanced downstream signaling.

Taken together, this study might help to design therapeutical agents, such as the Fab fragments of UCHT1, to specifically enhance tumor cell killing by γδ T cells while preventing unspecific activation of all T cells.

## Ethics Statement

Informed consent for the performed studies was obtained from the donors in accordance with the Declaration of Helsinki and Institutional Review Board approval from the University of Freiburg Ethics Committee (412/9).

## Author Contributions

CJ performed the killing assays as well as the CD69 and CD25 up-regulation experiments. CJ and AM prepared and tested the Fab fragments and performed the signaling assays. CJ, KR, H-HO, DW, and AM expanded human γδ T cells. PW, CJ, and FH performed the PLA experiments and JH performed the ELISA. PF, SM, SP, and WS conceived the experiments. CJ, WS, PW, DW, PF, SM, and SP wrote or edited the manuscript.

## Conflict of Interest Statement

The authors declare that the research was conducted in the absence of any commercial or financial relationships that could be construed as a potential conflict of interest.
