# Supplementary material for: Anti-CD3 Fab Fragments Enhance Tumor Killing by Human γδ T Cells Independent of Nck Recruitment to the γδ T Cell Antigen Receptor
Source: Front Immunol. 2018 Jul 9;9:1579. doi: 10.3389/fimmu.2018.01579 (PMC6046647; doi:10.3389/fimmu.2018.01579)
Supplement: Supplementary file 1 [file data_sheet_1.PDF]

Anti-CD3 Fab fragments enhance tumor killing by human γδ T cells independent of Nck recruitment to the γδ TCR

Claudia Juraske, Piyamaporn Wipa, Anna Morath, Jose Villacorta Hidalgo, Frederike A. Hartl, Katrin Raute, Hans-Heinrich Oberg, Daniela Wesch, Paul Fisch, Susana Minguet, Sutatip Pongcharoen, Wolfgang W. Schamel

SUPPLEMENTAL DATA

Supplemental Figure 1 page 1
Supplemental Figure 2 page 2
Supplemental Figure 3 page 2
Supplemental Figure 4 page 2
Supplemental Figure 5 page 3

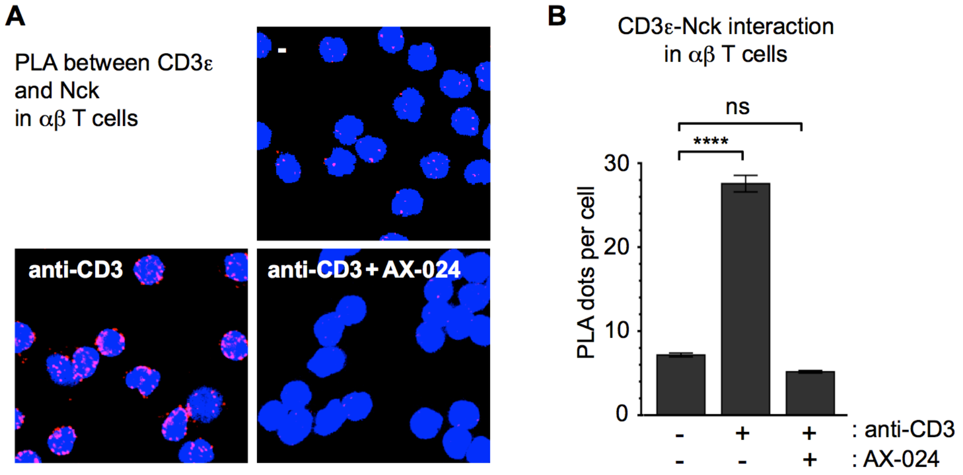

SUPPLEMENTAL FIGURE 1 | AX-024 reduces the PLA signal in anti-CD3 stimulated αβ T cells. (A) Close proximity between the TCR and Nck was detected by *in situ* PLA. Purified primary αβ T cells from human blood were either left untreated (-), or stimulated with 5 µg/ml anti-CD3 (OKT3) in the absence or presence of 10 nM AX-024 at 37°C for 5 min. PLA was performed as in figure 2. **(B)** The corresponding quantification of the red PLA dots and the mean ± SEM is displayed; Statistics was done by two-tailed t test. For each condition approx. 800 cells were analysed. Three independent experiments were performed (n=3). This experiment shows that PLA can be used to detect a block in the interaction of SH3.1(Nck) with the TCR in αβ T cells.

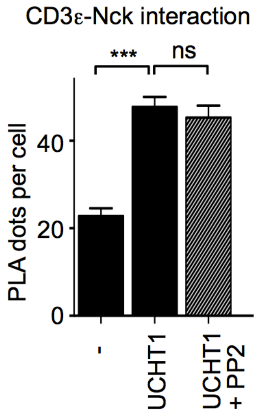

SUPPLEMENTAL FIGURE 2 | Inhibition of CD3 phosphorylation by PP2 does not prevent binding of Nck to the γδ TCR. Close proximity between the TCR and Nck was detected by *in situ* PLA. Zoledronate expanded γδ T cells were either left untreated or treated with 5 µg/ml UCHT1 in the absence or presence of 20 µM PP2 at 37°C for 5 min. PLA was performed and quantified as in figure 2. For each condition 200 cells were analyzed (n=1). This experiment shows that CD3 phosphorylation is not necessary for the recruitment of Nck to the γδ TCR.

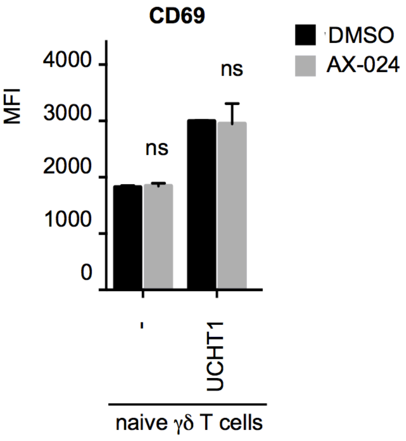

SUPPLEMENTAL FIGURE 3 | In naive γδ T cells CD69 up-regulation is not inhibited by AX-024. Freshly prepared peripheral blood mononuclear cells of a healthy donor were stimulated without (-) or with 5 µg/ml UCHT1 at 37°C and 5% CO_2 for 18 hours. Subsequently, cells were fixed and stained with an anti-CD69 antibody. Afterwards, cells were permeabilized and stained with anti-CD3 and anti-γ/δTCR antibodies. Fluorescence intensities were quantified by flow cytometry and the mean fluorescence intensity (MFI) is displayed. Data were analyzed as in figure 7 (n=2). This experiment shows that UCHT1-induced upregulation of CD69 is independent of Nck recruitment to the TCR in naïve γδ T cells.

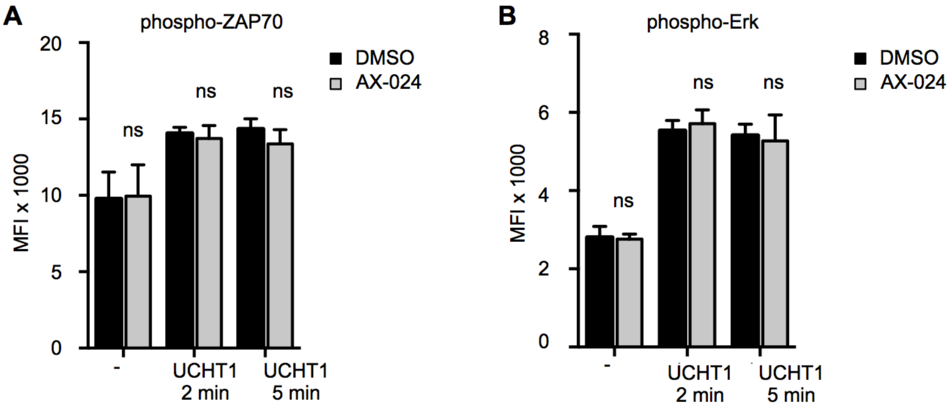

SUPPLEMENTAL FIGURE 4 | Phosphorylation of ZAP70 and Erk is not inhibited by AX-024 treatment. Freshly prepared peripheral blood mononuclear cells were stimulated without (-) or with 10 µg/ml UCHT1 at 37°C for 2 or 5 min. Subsequently, cells were fixed, permeabilised and stained with anti-γ/δTCR and (**A**) anti-phospho-ZAP70 or (**B**) anti-phospho-Erk antibodies. Fluorescence intensities were quantified by flow cytometry and the mean fluorescence intensity (MFI) of the phospho-protein in γδ T cells is displayed. Data were analyzed as in figure 7 (n=2). This experiment shows that phosphorylation of ZAP70 and Erk does not require the Nck-γδ TCR interaction.

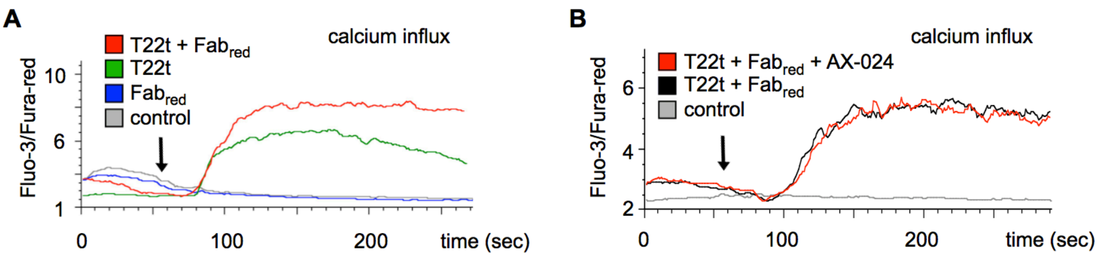

SUPPLEMENTAL FIGURE 5 | Calcium signaling is enhanced by Fab$_{red}$ fragments and is not inhibited by AX-024 treatment. (A and **B)** The calcium influx measurements were performed as in figures 8B and 8C, with the only difference that Fab$_{red}$ instead of Fab fragments were used. This experiment demonstrates that T22 tetramer-induced calcium signaling is enhanced by Fab$_{red}$ fragments and that this calcium response does not require Nck recruitment to the G8 γδ TCR.
